# Histological and transcriptional characterization of the pancreatic acinar tissue in type 1 diabetes

**DOI:** 10.1136/bmjdrc-2020-002076

**Published:** 2021-05-23

**Authors:** Louise Granlund, Anders Hedin, Miriam Wahlhütter, Peter Seiron, Olle Korsgren, Oskar Skog, Marcus Lundberg

**Affiliations:** 1 Department of Immunology, Genetics and Pathology, Uppsala Universitet, Uppsala, Sweden; 2 Department of Clinical Chemistry and Transfusion Medicine, Institute of Biomedicine, University of Gothenburg, Goteborg, Sweden

**Keywords:** pancreas, diabetes mellitus, type 1, transcription, genetic, pathology

## Abstract

**Introduction:**

Despite a reduced function and volume of the exocrine pancreas in type 1 diabetes, the acinar cells remain understudied in type 1 diabetes research. The hypothesis of this study is that the acinar tissue is altered in subjects with type 1 diabetes compared with subjects without diabetes.

**Research design and methods:**

The cell density, expression of digestive enzymes, and transcriptome of acinar tissue at varying distances from islets were analyzed using histology, immunostaining, and AmpliSeq RNA sequencing of laser capture microdissected tissue. Pancreases examined were from organ donors with or without type 1 diabetes.

**Results:**

We demonstrate preserved acinar nuclei density and find no support of acinar atrophy in type 1 diabetes. Staining for digestive enzymes (amylase, lipase, and trypsin) demonstrated an evenly distributed expression in the exocrine parenchyma; although occasional amylase-negative regions appeared in tissue that had been formalin-fixed and paraffin-embedded, this phenomenon was not evident in frozen tissue. Gene set enrichment analysis of whole transcriptome data identified transcriptional alterations in type 1 diabetes that were present in the acinar tissue independent of the distance from islets. Among these, the two most enriched gene sets were *Myc Targets V2* and *Estrogen Response Early*.

**Conclusion:**

Taken together, these new data emphasize the involvement of the entire pancreas in type 1 diabetes pathology. The alteration of the gene sets *Myc Targets V2* and *Estrogen Response Early* is a possible link to the increased incidence of pancreatic cancer in type 1 diabetes.

**Significance of this study**

**What is already known about this subject?**

- Type 1 diabetes has recently been recognized to involve the entire pancreas with reports of reduced pancreas volume and exocrine dysfunction.

**What are the new findings?**

- We find no histological support of acinar atrophy in acinar type 1 diabetic tissue but identify enriched gene sets independent of the distance from islets.

**How might these results change the focus of research or clinical practice?**

- This provides further evidence of the involvement of the entire pancreas in type 1 diabetes pathology and enhance our understanding of the pathology of the disease.

## Introduction

Classically described as a disease affecting the beta cells, type 1 diabetes has recently been recognized to involve the entire pancreas^1^ with reports of reduced pancreas volume^2–8^ and exocrine dysfunction.^9–14^ It has been proposed that beta-cell loss in type 1 diabetes is responsible for the reduced pancreas size and function of acinar tissue due to loss of trophic effects exerted by insulin.^15^ However, others debate that exocrine damage precedes type 1 diabetes onset,^10 16^ suggesting that acinar volume/function loss is caused by other factors than an indirect effect of beta-cell loss.

Acinar cells adjacent to islets devoid of insulin have been described by Foulis and Stewart^17^ to have severe atrophy, be smaller, and contain fewer zymogen granules. However, this association between acinar tissue atrophy and insulin-negative islets was not confirmed by Löhr and Klöppel^5^ who conversely reported a general atrophy of the acinar tissue in type 1 diabetes. Both studies were performed on autopsy material where the pancreas is exposed to various grades of autolysis. A more recent paper reported patches of amylase-negative regions in the exocrine pancreas as a common finding in non-diabetic subjects, with a reduced frequency in subjects with type 1 diabetes.^18^ Furthermore, Wright *et al*^19^ reported a reduced pancreas volume in type 1 diabetes subjects due to reduced acinar cell numbers (with unaltered acinar cell size). Gepts^20^ reported in 1965 that acinar tissue in proximity to ducts is atrophic. The acinar tissue in type 1 diabetes has not been further described with molecular methods.

The hypothesis tested in this study was that the acinar tissue is altered, on a histological and transcriptional level, in subjects with type 1 diabetes compared with subjects without diabetes. Additionally, we aimed to investigate whether such differences are particularly pronounced in exocrine regions in close proximity to islet tissue.

## Research design and methods

### Human pancreatic specimens

Pancreases from heart-beating organ donors treated for organ transplantation were procured through the Nordic Network for clinical Islet Transplantation (https://nordicislets.medscinet.com/en.aspx). The pancreases were dissected, and biopsies were immediately fixed in 4% paraformaldehyde (PFA) or snap-frozen in liquid nitrogen and subsequently stored at −80°C. At the time of the study, the biobank contained biopsies from 12 donors with long-standing type 1 diabetes, with a disease duration of years, and more than 2000 non-diabetic donors. Frozen biopsies from all 12 donors with long-standing type 1 diabetes and non-diabetic donors that were age-matched, sex-matched, and BMI-matched to the type 1 diabetes donors were screened for CD45 infiltration and tissue quality.

Briefly, a 10 µm section cut with a cryostat (Leica, CM1860 UV, at −20°C) was double-stained for synaptophysin and CD45 (antibodies summarized in online supplemental table S1) using the EnVision G/2 Doublestain System, Rabbit/Mouse (diaminobenzidine (DAB)+/Permanent Red, Dako, Jena, Germany) and counterstained with hematoxylin (cat. nr. 01800, Histolab Products, Gothenburg, Sweden). Biopsies from three of the type 1 diabetes donors had an immune cell infiltration in exocrine tissue that was so pronounced that it would have substantially influenced the transcriptome analysis of acinar cells and were therefore excluded from further analysis. Biopsies from an additional two type 1 diabetes donors were excluded as they consisted mainly of adipose and fibrotic tissue, with a sparse number of islets and acinar tissue that could not be extracted with the utilized laser capture microdissection (LCM) protocol. Biopsies from the non-diabetic donors were similarly assessed for immune cell infiltration and tissue quality, excluding biopsies on the same basis as the type 1 diabetes donors, resulting in biopsies from eight donors being included for further examination. The characteristics of the 15 donors that passed screening and were included in the study are shown in online supplemental table S2. Five of the seven included donors with type 1 diabetes were also included in a previous study of islet characteristics.^21^ Formalin-fixed paraffin-embedded (FFPE) biopsies were histologically evaluated from the same donors.

10.1136/bmjdrc-2020-002076.supp1 Supplementary data

### Sectioning strategy of biopsies and immunofluorescent staining of endocrine cells

The frozen biopsies that passed the screening were sectioned in consecutive series (10 µm per section). Every second section was prepared for LCM by mounting on Arcturus PEN Membrane Glass Slides (LCM0522, Thermo Fisher Scientific, Waltham, Massachusetts, USA) and stored at −80°C until microdissection, generating a total of 10 LCM sections per biopsy. Of the remaining sections, every second was used for triple immunofluorescence (IF, see below for further information) of insulin, glucagon, and somatostatin or saved for future analysis (online supplemental figure S1). Four sections for IF and five additional slides were collected, mounted on Superfrost Plus glasses (Menzel-Gläser, Braunschweig, Germany), and stored at −80°C. The sections intended for endocrine cell IF staining were fixed in 4% PFA for 15 min, blocked with 5% normal goat serum (Dako) in 1× TBS-Tween (EnVision FLEX Wash Buffer, 20×, Dako) for 60 min, and incubated with anti-insulin, anti-glucagon, and anti-somatostatin antibodies (online supplemental table S1) diluted in 5% goat serum in 1× TBS-Tween for 60 min. Nuclei were stained with 500 nM SYTOX Orange nucleic acid stain. Slides were scanned on a confocal microscope (LSM700, Zeiss, Oberkochen, Germany). The scans were used to locate islets and scattered endocrine cells. This in turn was used to define acinar islet-adjacent areas and areas lacking endocrine cells that were microdissected on consecutive PEN membranes. The methodology was designed to only extract and study areas adjacent to insulin-negative islets in type 1 diabetes subjects, but it was discovered that all islets were insulin negative and therefore no selection was necessary.

### Immunohistochemistry and IF of acinar cells

Sections (6 µm) from each FFPE tissue sample block were processed and labeled using a standard technique. The sections were stained for either synaptophysin or the exocrine enzymes amylase, trypsin, or lipase. All antigens were unmasked by heat-induced antigen retrieval using EDTA according to the manufacturer’s instructions (Dako). Primary antibodies (online supplemental table S1) against synaptophysin, trypsin, lipase, or amylase were applied and thereafter visualized using Dako EnVision and DAB-based substrate. Three different antibodies targeting amylase were used in non-diabetic samples to validate possible findings, and thereafter one of the antibodies (HPA045394) was utilized in type 1 diabetes samples. Sections were counterstained with hematoxylin (Histolab) and analyzed using a light microscope (Leica DM2000 LED, Wetzlar, Germany) or Aperio ScanScope system (Aperio Technologies, Oxford, UK). The nucleus density (nuclei/mm^2^) was evaluated with assistance of ImageJ software on images obtained at 20× magnification. A minimum of five areas of two acinar regions, located either in direct proximity to islets (0–50 µm from islets, Exo1) or far from islets, defined as regions in areas where islets were not present, a minimum of 100 µm from islets (Exo3), were selected at random, and the number of nuclei were counted. On average, 811 nuclei were counted in every region of Exo1 and Exo3, respectively. The nuclei density was examined in all donors (online supplemental table S2), including the three type 1 diabetic donors that were excluded from the LCM analysis because of extensive immune cell infiltration in exocrine tissue. During the examination of the amylase staining pattern, acinar cell origin was verified by positive staining of trypsin and lipase (data not shown).

Frozen biopsies (online supplemental table S2) were sectioned (10 µm) and fixed in 4% PFA for 15 min and then blocked with 5% normal goat serum (Dako) in 1× TBS-Tween (Dako) for 30 min. Primary antibodies against amylase and glucagon (online supplemental table S1) were diluted in 5% goat serum in 1× TBS-Tween, applied and incubated for 60 min after which the secondary antibody for amylase (online supplemental table S1) were diluted in 5% goat serum in 1× TBS-Tween, applied and incubated for 60 min. Nuclei were stained with 500 nM SYTOX Orange nucleic acid for 10 min. The sections were imaged on a confocal microscope (LSM700, Zeiss).

### Laser capture microdissection

The samples were removed from −80°C, thawed and dehydrated using the following protocol; 75% EtOH for 30 s at −20°C, followed by 95% EtOH for 1 min, 100% EtOH for 1 min, and xylene for 4 min at room temperature (KIT0401, HistoGene LCM alcohol kit, Thermo Fisher Scientific). After drying, the membrane glass slides were mounted on an Arcturus XT LCM system (Thermo Fisher Scientific). Islets were identified based on islet autofluorescence (online supplemental figure S2) and verified by the scanned IF slides. By identifying the islets, three different groups of exocrine tissue could be defined and acquired. Exocrine tissue adjacent to islets was defined as tissue within a 50 µm distance from islet tissue (Exo1), exocrine tissue at a distance between 50 and 100 µm from the closest islet (Exo2), and exocrine tissue far from islets, defined as regions in areas where islets were not present, a minimum of 100 µm from the closest islet (Exo3) (online supplemental figure S2). The regions were captured on an Arcturus CapSure HS LCM Cap (LCM0215, Thermo Fisher Scientific). The cap was subsequently incubated in 20 µL of Buffer RLT Plus (Qiagen, Sollentuna, Sweden) with 1% beta-Mercaptoethanol in a heating block for 30 min at 42°C, lysing the tissue. The lysates were stored at −80°C until RNA extraction. Areas of the cut regions were noted.

### RNA extraction

The samples were brought to room temperature by short incubation at 37°C. All LCM-extracted samples were pooled for each respective tissue region for each donor. RNA was extracted with the RNeasy Plus Micro kit (Qiagen) according to the manufacturer’s protocol for purification of total RNA from microdissected cryosections. Samples were eluted with RNase-free water.

### AmpliSeq RNA sequencing

For each sample, total RNA was reverse-transcribed to cDNA using the Ion AmpliSeq Transcriptome Human Gene Expression Kit (Thermo Fisher Scientific). The acquired cDNA was amplified using the Ion AmpliSeq Transcriptome Human Gene Expression core panel and adaptors (Ion P1 Adaptor and Ion Xpress Barcode Adaptor) were ligated to the amplicons. Adaptor-ligated amplicons were purified using Agencourt AMPure XP reagents, eluted in amplification mix and amplified. Size selection and purification were conducted using Agencourt AMPure XP reagents. Sequencing of sample pools was then done in four separate runs using the Ion 550 Kit and the IonS5XL instrument (Thermo Fisher Scientific). Acquired reads were analyzed using the AmpliSeq RNA plugin in the Torrent Suite Server V.5.10.1. The reads were aligned to hg19 AmpliSeq Transcriptome ERCC V.1, quantifying expression data for 20 813 genes. One sample (Exo2 from donor T1D-7) produced low quality reads and were excluded from further data analysis.

### Statistical analysis

GraphPad Prism software (V.6.0h) was used for statistical analysis. The Mann-Whitney test was used to compare nuclei/mm^2^ between non-diabetic and type 1 diabetes donors, whereas the Wilcoxon test was used to compare nuclei/mm^2^ between Exo1 and Exo3 within the respective groups. A p value<0.05 was considered statistically significant.

#### Filtering

Data was analyzed using the edgeR R package (V. 3.28.1)^22 23^ starting from raw read counts. Genes with more than 10 counts per million in at least six samples were retained using the filterByExp function of edgeR.

#### Deconvolution analysis

Cell type proportions of the LCM-extracted bulk data were estimated with Multi-subject Single Cell (MuSiC) deconvolution using the R package MuSic (V. 0.1.1).^24^ The raw counts were analyzed with the E-MTAB-5061 human pancreas single-cell data as reference dataset (https://www.ebi.ac.uk/arrayexpress/experiments/E-MTAB-5061/).^25^ Results were visualized using ggplot2 (V. 3.3.0).

#### Normalization

Raw count normalization was performed using the trimmed mean of M values (TMM)^26^ method with the calcNormFactors- function.

#### Data structure

TMM-adjusted and log-normalized counts were used to visualize the data structure of the acinar tissue by principal component analysis (PCA) using the R- package PCAtools.^27^

#### Differential gene expression analysis

Differentially expressed genes (DEGs) between diabetic and non-diabetic donors for each acinar tissue region were analyzed using a generalized linear model and a quasi-likelihood test with the glmQLFit and glmTreat functions of edgeR. Genes differentially expressed in type 1 diabetes compared with non-diabetic subjects were assessed. False discovery rate (FDR)-adjusted p values were calculated using the Benjamini-Hochberg method in the topTags function in edgeR. Criteria for differential expression was FDR-adjusted p value <0.05 while testing for an absolute log fold change ≥log2(1.2).

#### Functional enrichment analysis

Competitive gene set testing was conducted with CAMERA (Correlation Adjusted MEan RAnk test)^28^ using the camera function in edgeR^22 23^ and the MSigDB Hallmark set.^29^ A gene set was considered significantly enriched if the FDR-adjusted p value <0.25.

#### Data and resource availability

The datasets generated and analyzed during the current study are available in the GEO repository, GSE162689. No applicable resources were generated or analyzed during the current study.

## Results

### Similar acinar cell density in pancreases from donors with or without type 1 diabetes regardless of the distance to islets

**Figure 1:**
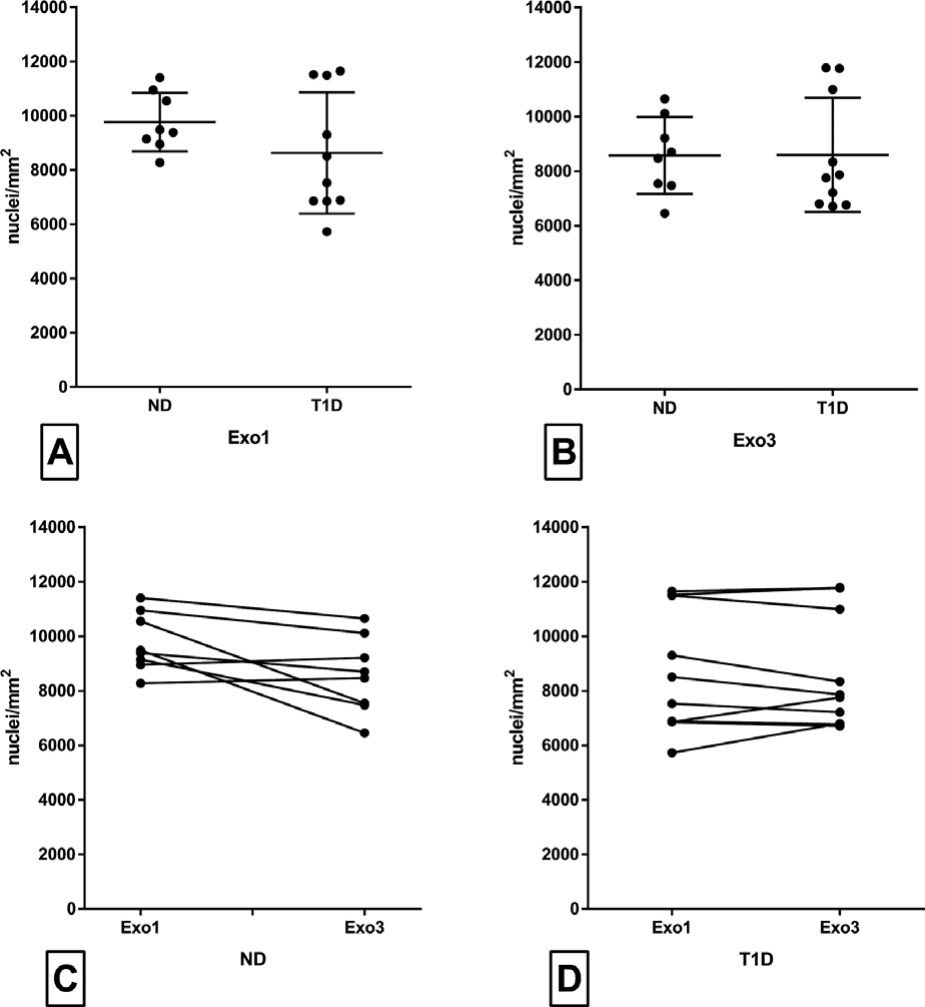
Density of acinar cells (nuclei/mm^2^) was manually examined on formalin-fixed paraffin-embedded sections with immunohistochemical staining for synaptophysin and hematoxylin. All type 1 diabetic islets had already previously been shown to be insulin-negative by immunofluorescent staining. Regions adjacent to islets (Exo1) and regions in areas where islets were not present, a minimum of 100 µm from islets, (Exo3) were examined. Acinar density in ND was compared with T1D in Exo1 (A) and Exo3 (B). Acinar density in Exo1 was compared with Exo3 in ND (C) and T1D (D). Each dot represents an individual sample. In (A) and (B), the line illustrated the mean value, and the error bars illustrate SD. In (C) and (D), interconnected dots illustrate paired samples from the same donor. The Mann-Whitney test was used between groups in (A) and (B) and the Wilcoxon test was used in (C) and (D). ND, non-diabetic subjects; T1D, type 1 diabetic subjects.

All islets in donors with type 1 diabetes were insulin-negative, although a few insulin-positive cells were found scattered in the exocrine parenchyma in 3/7 donors with type 1 diabetes (online supplemental table S2 and figure S2). The nuclei density (nuclei/mm^2^) in acinar tissue was unaltered in donors with type 1 diabetes compared with non-diabetic donors, both in regions directly adjacent to islet tissue (Exo1) and in regions far away from the closest islet (Exo3) (figure 1A, B), although the interindividual difference was large. The mean acinar cell nuclei density was marginally higher in Exo1 than in Exo3 in the pancreas of non-diabetic subjects (figure 1C); however, no difference could be determined between Exo1 and Exo3 within the pancreas of type 1 diabetic subjects (figure 1D).

### Amylase-negative acinar regions were found in FFPE but not in frozen tissue

**Figure 2:**
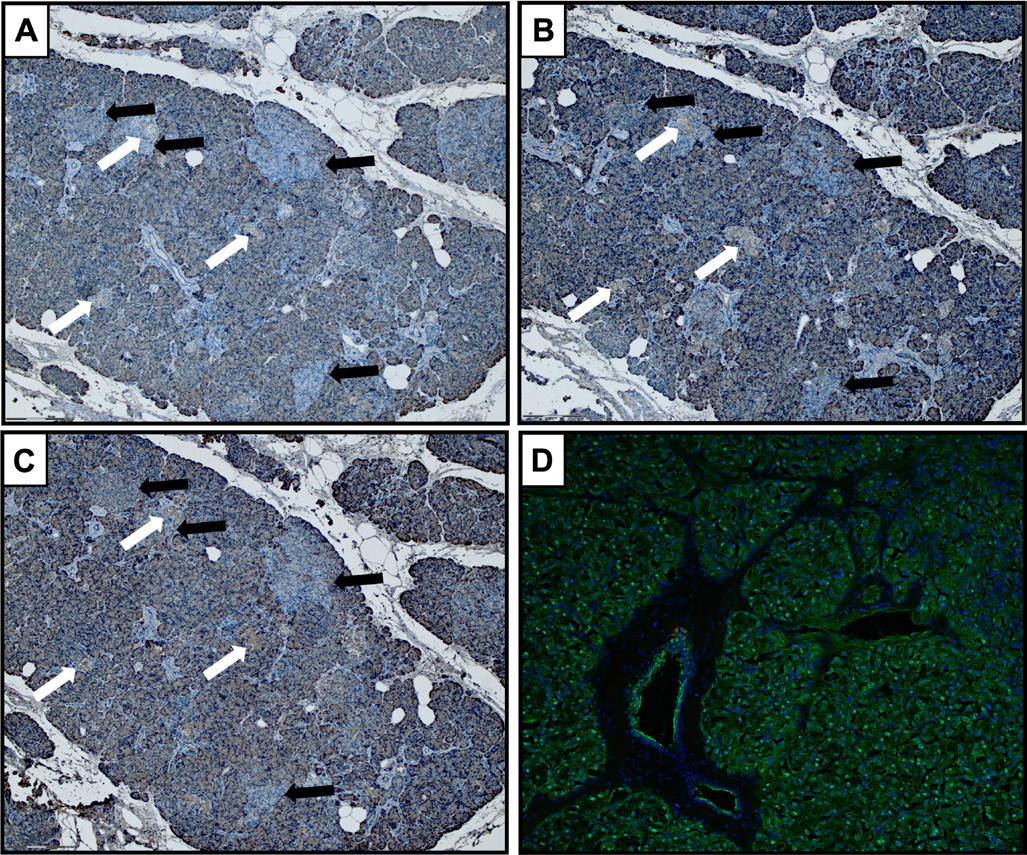
Amylase-negative acinar areas (black arrows) were observed in a subset of donors, with or without type 1 diabetes, stained with three different antibody clones against amylase ((A), clone HPA045394; (B), clone LSB12950; (C), clone AB21156) on FFPE tissue. No amylase-negative acinar areas were found using IF on frozen tissue from the same donors (D). In FFPE sections, the amylase-negative areas were found both adjacent and non-adjacent to islets (white arrows). FFPE, formalin-fixed paraffin-embedded.

Amylase-negative acinar regions (figure 2A–C), as described by Kusmartseva *et al*,^18^ were found by immunohistochemistry (IHC) with three different antibodies against amylase on FFPE tissue from 3/7 donors with type 1 diabetes and 1/8 non-diabetic controls. The same phenomenon was not evident by IF of frozen sections from the same donors, where an even expression of amylase was seen in acinar cells throughout the exocrine parenchyma (figure 2D).

### No clear clustering of samples based on disease status

**Figure 3:**
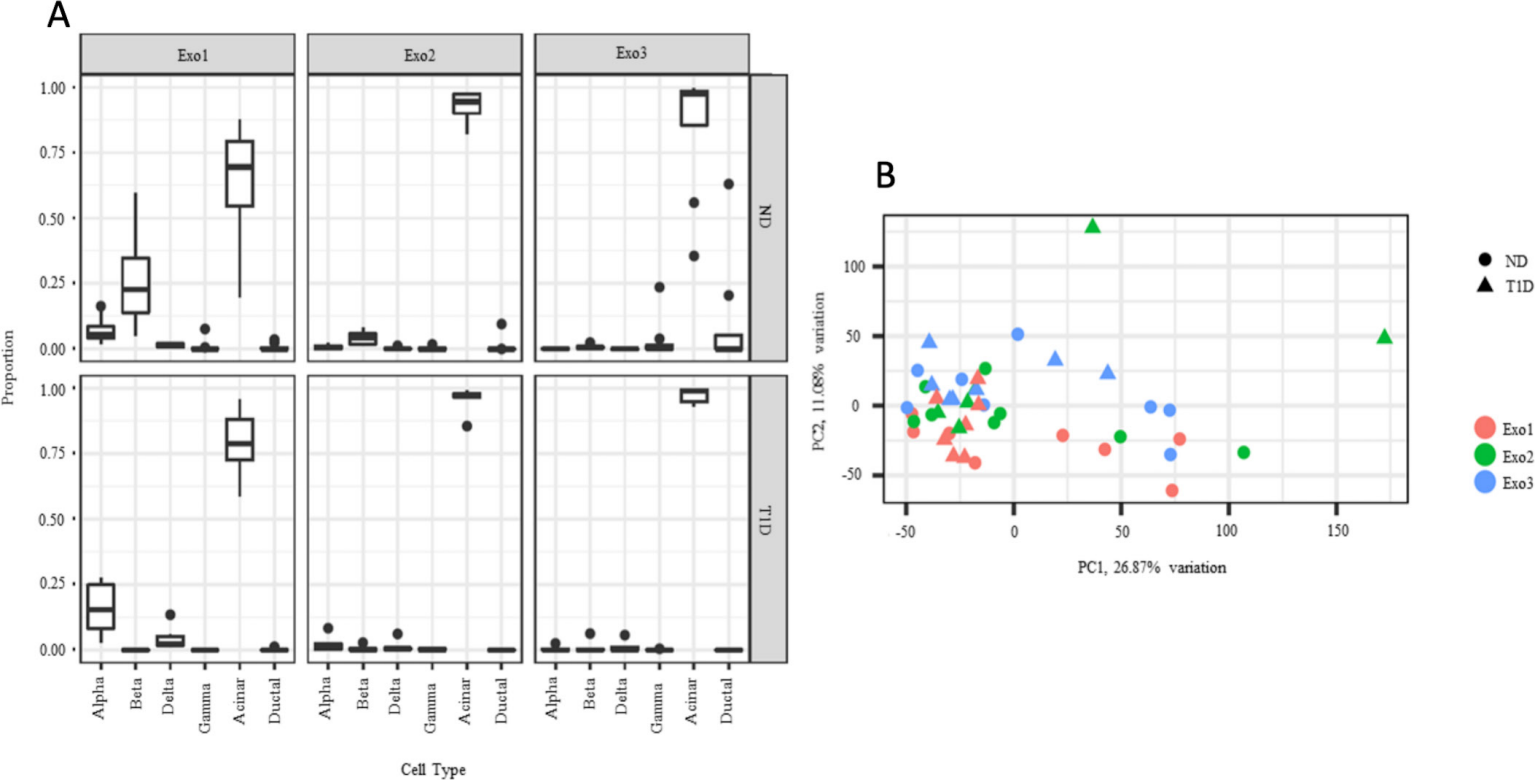
(A) Multi-subject Single Cell (MuSiC) utilizes cell type-specific gene expression from single-cell RNA sequencing data to characterize cell type composition from bulk data. The figure illustrates deconvolution of the bulk data into alpha, beta, gamma, delta, acinar, and ductal cells in the different tissues, divided by disease status as performed by MuSiC deconvolution. Proportions sum to one per sample, and the data is illustrated in a Tukey boxplot. (B) The 25% most variable genes of exocrine tissue were used for principal component analysis. Each point corresponds to a sample plotted by PC1 and PC2. PC1 and PC2 describe 26.9% and 11.1% of the exocrine variation, respectively. Pink circles, ND Exo1; green circles, ND Exo2; blue circles, ND Exo3; pink triangles, T1D Exo1; green triangles, T1D Exo2; blue triangles, T1D Exo3. ND, non-diabetic subjects; T1D, type 1 diabetic subjects.

To assess the presence of contaminating islet cells in the samples microdissected from different exocrine regions, cell type deconvolution of the transcriptome data was done. Samples extracted from the regions just adjacent to islet tissue (Exo1) were partly contaminated by islet tissue, both in type 1 diabetic and non-diabetic subjects, whereas samples extracted from regions further separated from islets (Exo2 and Exo3) had minimal contamination by islet tissue (figure 3A). A PCA of the 25% most variable genes across exocrine tissue shows no apparent clustering based on exocrine region or disease status (figure 3B).

### Few DEGs were found in exocrine tissue of subjects with and without type 1 diabetes

**Figure 4:**
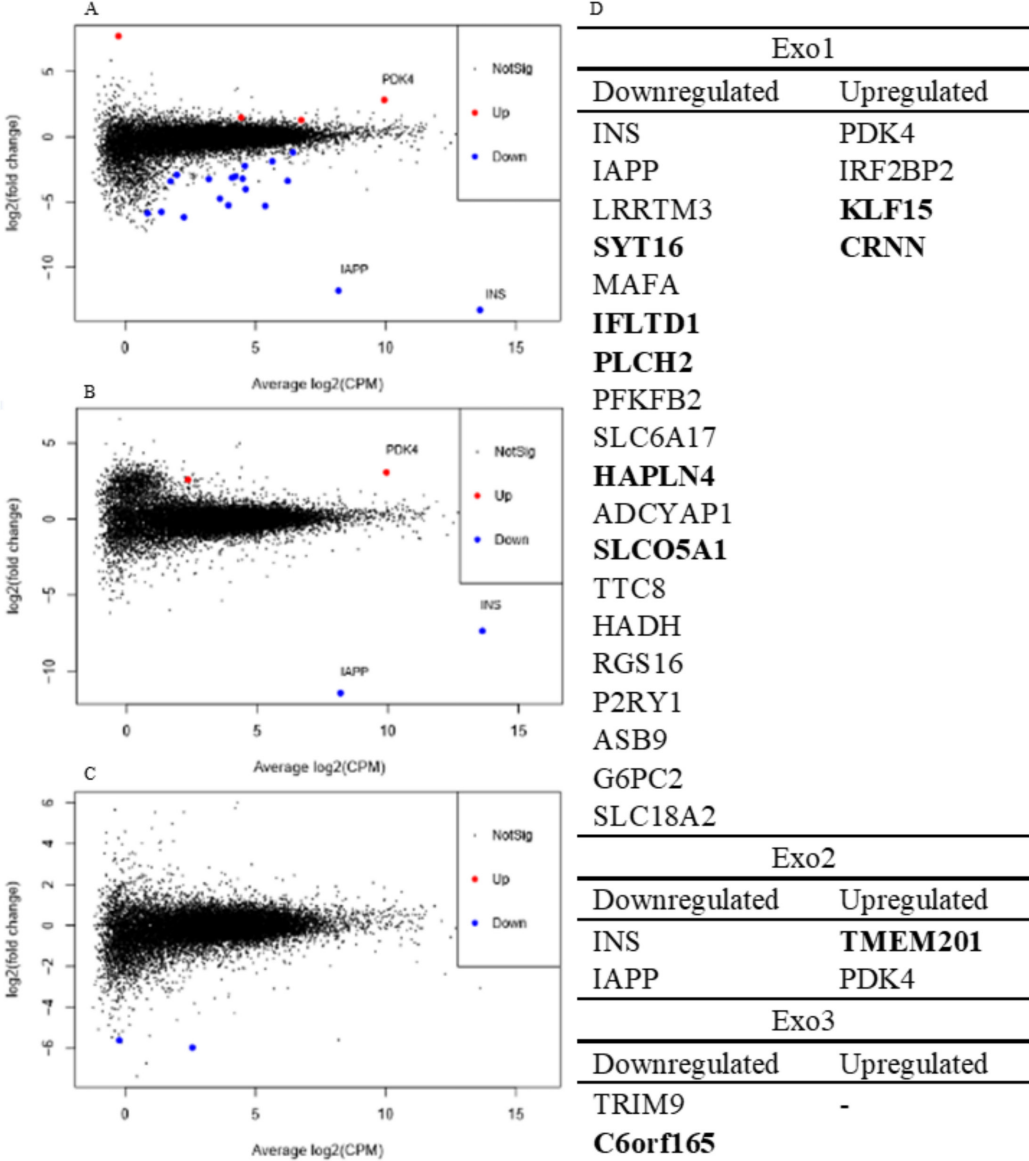
(A, B, C) Mean difference plots of genes, in type 1 diabetes compared with non-diabetic tissue of Exo1 (A), Exo2 (B), and Exo3 (C). Log2(fold change) was compared with average log2(CPM) of all type 1 diabetic and non-diabetic samples. Differentially expressed genes (DEGs) at an FDR-adjusted p value <0.05 and an absolute log fold change ≥log2(1.2) are highlighted. (D) DEGs at an FDR-adjusted p value <0.05 and an absolute log fold change ≥log2(1.2) in subjects with type 1 diabetes when compared with non-diabetic subjects in exocrine regions. CPM, counts per million; Exo1, region adjacent to islet; Exo2, region 50–100 µm from islet; Exo3, region in an area where islets were not present, a minimum of 100 µm from islets; FDR, false discovery rate.

In total, 29 DEGs in type 1 diabetes were found in exocrine regions (figure 4). Among these, 19 DEGs were overlapping with genes reported to be islet-enriched in at least one of four previous reports.^25 30–32^ The DEGs not overlapping with genes reported to be islet-enriched are marked in bold letters in figure 4D. Most of these were found in Exo1, in which 19 genes were downregulated and four upregulated in type 1 diabetes (figure 4A, D). In Exo2, two genes were downregulated and two genes were upregulated (figure 4B, D). In Exo3, two genes, *TRIM9* and *c6orf165*, were downregulated in type one diabetes (figure 4C, D).

### Similar gene sets were enriched in type 1 diabetes in all three exocrine regions

**Figure 5:**
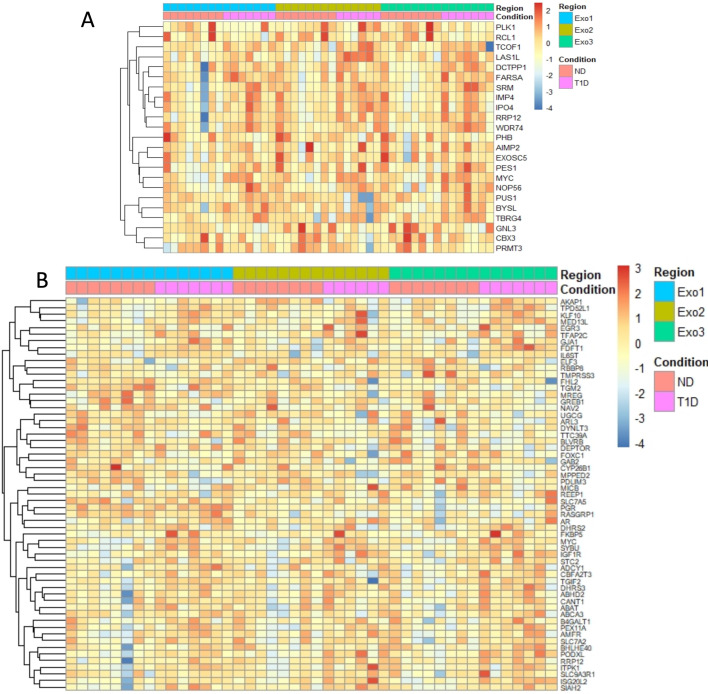
(A) Heatmap of islet expression of genes in HALLMARK_MYC_TARGETS_V2 with a differential expression between ND and type 1 diabetic subjects in any exocrine region, positive or negative, relative to a threshold of log2(1.2) using a cutoff of FDR<0.70 and the glmTREAT function in edgeR. Color indicates z-score calculated from log2(TMM-CPM normalized counts), as indicated by the color key. (B) Heatmap of islet expression of genes in HALLMARK_ESTROGEN_RESPONSE_EARLY with a differential expression between ND and type 1 diabetic subjects in any exocrine region, positive or negative, relative to a threshold of log2(1.2) using a cutoff of FDR<0.70 and the glmTREAT function in edgeR. Color indicates z-score calculated from log2(TMM-CPM normalized counts), as indicated by the color key. FDR, false discovery rate; ND, non-diabetic subjects; T1D, type 1 diabetic subjects; TMM, trimmed mean of M values.

MSigDB Hallmark gene sets found to be enriched in type 1 diabetes in the different exocrine regions are displayed in table 1. Several gene sets were significantly enriched in all exocrine regions despite the limited number of DEGs. The two most enriched gene sets in type 1 diabetes for all exocrine regions (ie, Exo1, Exo2, and Exo3, respectively) were *myc targets* v2 (figure 5A) and *estrogen response early* (figure 5B), although the later was not significantly enriched in Exo2.

**Table 1:** Gene sets found to be differentially expressed (FDR<0.25) in exocrine tissue between non-diabetic and type 1 diabetes subjects

Gene set	N of genes	P value	FDR
**Upregulated gene sets Exo1**			
HALLMARK_ESTROGEN_RESPONSE_EARLY	171	0.000668	0.0277
HALLMARK_MYC_TARGETS_V2	58	0.00111	0.0277
HALLMARK_P53_PATHWAY	172	0.00174	0.0290
HALLMARK_ADIPOGENESIS	180	0.00238	0.0297
HALLMARK_TNFA_SIGNALING_VIA_NFKB	172	0.00471	0.0431
HALLMARK_WNT_BETA_CATENIN_SIGNALING	35	0.00517	0.0430
HALLMARK_MYC_TARGETS_V1	192	0.00794	0.0567
HALLMARK_UNFOLDED_PROTEIN_RESPONSE	106	0.0125	0.0694
HALLMARK_CHOLESTEROL_HOMEOSTASIS	68	0.0125	0.0694
HALLMARK_HYPOXIA	160	0.0156	0.0777
HALLMARK_HEME_METABOLISM	161	0.0171	0.0777
HALLMARK_UV_RESPONSE_UP	136	0.0230	0.0959
HALLMARK_XENOBIOTIC_METABOLISM	162	0.0361	0.139
HALLMARK_FATTY_ACID_METABOLISM	141	0.0509	0.182
HALLMARK_PROTEIN_SECRETION	95	0.0672	0.210
**Downregulated gene sets Exo1**			
HALLMARK_ALLOGRAFT_REJECTION	149	0.0671	0.210
**Upregulated gene sets Exo2**			
HALLMARK_MYC_TARGETS_V2	58	0.00347	0.174
**Downregulated gene sets Exo2**			
–	–	–	–
**Upregulated gene sets Exo3**			
HALLMARK_MYC_TARGETS_V2	58	0.000386	0.0181
HALLMARK_ESTROGEN_RESPONSE_EARLY	171	0.000722	0.0181
HALLMARK_UNFOLDED_PROTEIN_RESPONSE	106	0.00239	0.0398
HALLMARK_ADIPOGENESIS	180	0.00408	0.0409
HALLMARK_MYC_TARGETS_V1	192	0.00409	0.0409
HALLMARK_TNFA_SIGNALING_VIA_NFKB	172	0.00535	0.0446
HALLMARK_HEME_METABOLISM	161	0.0112	0.0705
HALLMARK_WNT_BETA_CATENIN_SIGNALING	35	0.0137	0.0705
HALLMARK_HYPOXIA	160	0.0138	0.0705
HALLMARK_FATTY_ACID_METABOLISM	141	0.0165	0.0705
HALLMARK_PROTEIN_SECRETION	95	0.0167	0.0705
HALLMARK_XENOBIOTIC_METABOLISM	162	0.0169	0.0705
HALLMARK_P53_PATHWAY	172	0.0202	0.0779
HALLMARK_UV_RESPONSE_UP	136	0.0253	0.0903
HALLMARK_OXIDATIVE_PHOSPHORYLATION	177	0.0472	0.1574
HALLMARK_GLYCOLYSIS	169	0.0526	0.164
**Downregulated gene sets Exo3**			
–	–	–	–

FDR, false discovery rate.

## Discussion

In this study, we found no difference in acinar nuclei density in type 1 diabetes compared with ND controls, contrasting with the general atrophy of acinar tissue described previously by Foulis and Stewart^17^ and Löhr and Klöppel.^5^ Both of these previous reports were based on studies of autopsy samples where autolysis must be considered as another possible explanation for the findings. A more recent paper by Wright *et al*^19^ reported unaltered acinar cell size in adult subjects with long-standing type 1 diabetes (duration 2–48 years), in line with our findings, and attributed the reduced pancreas volume in subjects with type 1 diabetes to a reduced number of acinar cells.

Kusmartseva *et al*^18^ recently reported patches of amylase-negative regions in the exocrine pancreas as a common finding in non-diabetic subjects with a reduced frequency in subjects with type 1 diabetes. Here, amylase-negative areas were found in only one non-diabetic subject and three type 1 diabetic subjects. However, the pattern was only seen with IHC on FFPE biopsies and could not be replicated with IF on frozen tissue sections. A possible interpretation is that the phenomenon is very local, appearing in some lobules but not others. An alternative explanation is that the amylase-negative regions are artifacts of formalin fixation and paraffin embedding.

In light of the exocrine dysfunction often reported in subjects with type 1 diabetes,^9–13 18^ we hypothesized that this would be reflected by alterations in the acinar transcriptome. Although the global expression profiles of the samples did not cluster by disease status, 23 type 1 diabetes DEGs were discovered in exocrine regions adjacent to the islets (Exo1). However, as MuSiC analysis revealed that this region was partly contaminated by islet cells, it is likely that at least some of these 23 DEGs are explained by the loss of beta-cells in type 1 diabetes. Indeed, some genes known to be beta-cell specific, such as INS and IAPP, were among the DEGs in Exo1. In the exocrine regions further away from islet tissue (Exo2 and Exo3), that were less affected by islet cell contamination, only few DEGs were detected. Despite the modest number of DEGs, several gene sets were found to be significantly enriched in the acinar regions using gene set enrichment analysis. CAMERA was used to adjust for co-variation of prominent genes due to contamination, which may explain why the list of gene sets most enriched in Exo1 was similar to the list in Exo3, which was not contaminated by endocrine cells. These lists contained gene sets such as *Myc Targets V2*, *Estrogen Response Early*, *Unfolded Protein Response*, *Adipogenesis,* and *TNFA signalling via NFKB*. The same gene sets were also present among the top ranked in Exo2 although not statistically significant. In summary, this suggests that there were a general transcriptional alteration present in acinar type 1 diabetes tissue that did not depend on the distance from islets. As illustrated in the heatmaps of the most enriched pathways *Myc Targets V2* and *Estrogen Response Early*, small expression alterations in several genes most likely explain the enrichment of the gene sets, rather than large variations in a limited number of genes.

c-MYC is a proto-oncogene involved in many cellular processes such as cell growth, proliferation, and apoptosis. The transcription factor has been demonstrated to play a crucial role in pancreas development and differentiation but also the progression of pancreatic cancer.^33 34^ Diabetes and hyperglycemia have been linked to an increased risk of developing pancreatic cancer. In a recent study by Sato *et al,*^35^ a link is shown between hyperglycemia, the activation of MYC through STAT3, and the development of pancreatic cancer.^36^ Similarly, estrogen receptor-related pathways have been linked to an increased risk of developing pancreatic cancer.^37–39^ Alternatively, the activation of c-MYC and estrogen response genes could be a compensatory response to the reduced acinar volume^19^ to initiate proliferation of the tissue.

In conclusion, transcriptional alterations were present in the acinar tissue seemingly independent of the distance from islets. Two gene sets were enriched in all exocrine regions: *Myc Targets V2* and *Estrogen Response Early.* The alteration of these genes is a possible link to the increased incidence of pancreatic cancer in type 1 diabetes, which merits further investigation.

## Data Availability

Data are available in a public, open access repository. The datasets generated and analyzed during the current study are available in the GEO repository, GSE162689. No applicable resources were generated or analyzed during the current study.
