# Supplementary material for: Histological and transcriptional characterization of the pancreatic acinar tissue in type 1 diabetes
Source: BMJ Open Diabetes Res Care. 2021 May 23;9(1):e002076. doi: 10.1136/bmjdrc-2020-002076 (PMC8149357; doi:10.1136/bmjdrc-2020-002076)
Supplement: Supplementary data [file bmjdrc-2020-002076supp001.pdf]

Supplemental Material

Table S1. Antibodies used for immunohistochemistry (IHC) and immunofluorescence (IF). *Concentration used for triple staining with insulin, somatostatin and glucagon. †Concentration used for double staining with amylase and glucagon.

Antibody	Catalog #	Company	Concentration			Antigen Retrieval	Incubation Time
			IHC FFPE	IHC frozen	IF		
**Insulin**	565689	BD Bioscences	-	-	1:100	-	1 h
**Somatostatin**	566032	BD Bioscences	-	-	1:100	-	1 h
**Glucagon**	565891	BD Bioscences	-	-	1:100*/1:50†	-	1 h
**Sytox Orange**	S11368	Life Technologies	-	-	500 nM	-	10 min
**Synaptophysin**	DAK-SYNAP	Dako	1:100	1:140	-	pH 9	30 min
**CD45**	Clone 2B11 + PD7/26	Dako	-	1:75	-	-	30 min
**Amylase**	AB21156	Abcam	1:100	-	1:50	pH 6	30 min/1 h
**Amylase**	HPA045394-100UL	Sigma	1:600	-	-	pH 6	30 min
**Amylase**	LS-B12950-50	LSBio	1:300	-	-	pH 6	30 min
**Trypsin**	LS-B13760-200	LSBio	1:100	-	-	pH 6	30 min
**Lipase**	HPA062494-100UL	Sigma	1:800	-	-	pH 6	30 min
**AF488**	10729174	Fisher Scientific	-	-	1:500	-	1 h

Table S2. Clinical characteristics of the donors that passed the screening. All islets in donors with type 1 diabetes were insulin negative. “INS pos cells in exocrine tissue” refer to scattered insulin-positive cells surrounded by exocrine tissue as detected by immunofluorescent staining. +: present, -: not present. NDs: Non-diabetic subjects, T1Ds: Type 1 diabetic subjects, Na: Not available. * Biopsies used for LCM. †Biopsies used for IF stainings of amylase.

	**Donor No.**	**Age**	**Sex**	**BMI (kg/m2)**	**HbA1c, %, (mmol/mol)**	**Pancreas region frozen biopsies**	**Pancreas region FFPE biopsies**	**IA2A**	**GADA**	**INS pos cells in exocrine tissue**	**Nr of nuclei**		**Cause of death**
											**Exo 1**	**Exo 3**	
**NDs**	**ND-1**	13	M	16	5.8, 40	tail*, body†	tail	na	na	+	864	774	Celebral anoxia due to cardiac arrest
	**ND-2**	35	F	24.7	na	body*, head†	tail	-	-	+	1314	1014	Hypoxemia due to cardiac arrest
	**ND-3**	57	F	22.7	6.3, 45	tail*, body†	body	na	na	+	1280	700	Subarachnoid hemorrage
	**ND-4**	45	F	25.4	5.7, 38.8	tail*, tail†	head	-	-	+	722	928	Infarction in the cerebellum
	**ND-5**	21	M	28	5.7, 38.8	tail*, tail†	head	-	-	+	910	946	Head trauma
	**ND-6**	17	F	28.9	na	body*, body†	tail	na	na	+	1051	686	Traumatic subarachnoid hemorrage
	**ND-7**	63	M	24	5.8, 39.9	tail*, head†	head	-	-	+	896	825	Subarachnoid hemorrage
	**ND-8**	13	M	19.7	5.2, 33	tail*, tail†	head	-	-	+	883	828	Strangulation
**Mean±SD**		**33±18.8**		**23.7±4**							**990±196**	**838±110**	
**T1Ds**	**T1D-1**	16	M	21.9	na	tail*, body†	head	na	na	+	762	883	Traumatic subarachnoid hemorrage
	**T1D-2**	36	F	20.9	7.2, 55.2	tail*, tail†	body	na	na	+	461	624	Intracranial hemorrage
	**T1D-3**	60	F	23.9	8.2, 66.1	tail*, tail†	head	+	+	-	653	874	Subarachnoid hemorrage
	**T1D-4**	47	F	27.6	7.4, 57.4	body*, body†	head	-	-	+	415	509	Cardiac arrest
	**T1D-5**	24	M	27.5	8.3, 67.2	tail*, tail†	head	+	-	-	501	681	Trauma
	**T1D-6**	25	F	26.7	7.1, 54.1	tail*, body†	tail	-	-	-	925	675	Cerebral edema due to hypoglycemia
	**T1D-7**	65	M	24.2	na	tail*, tail†	body	na	na	-	1243	985	Trauma by fall
**Mean±SD**		**39±17.5**		**24.7±2.5**							**709±275**	**747±157**	

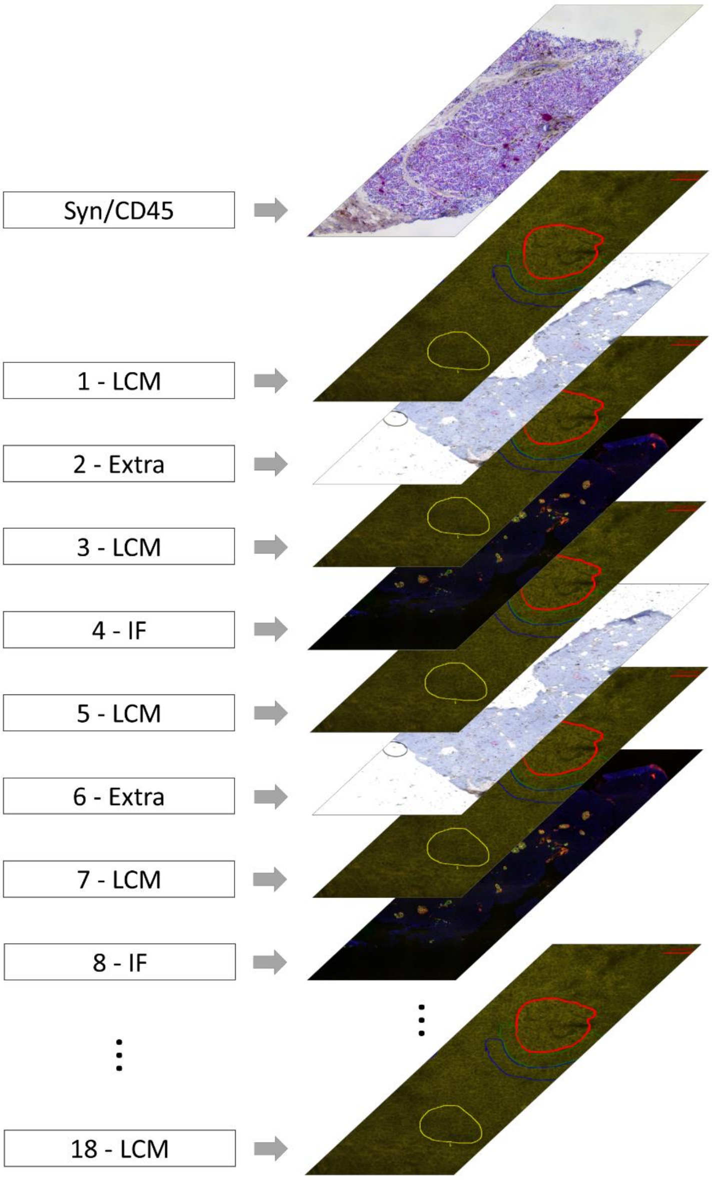

Figure S1. Strategy for consecutive sectioning prior to LCM. Frozen consecutive sections (10 µm) were mounted on either Arcturus PEN membrane glass slides for LCM or glass slides. The sections on glass slides were used for IF staining of insulin, glucagon and somatostatin, or saved for future analysis. Syn/CD45: synaptophysin/CD45, LCM: Laser Capture Microdissection, IF: Immunofluorescense.

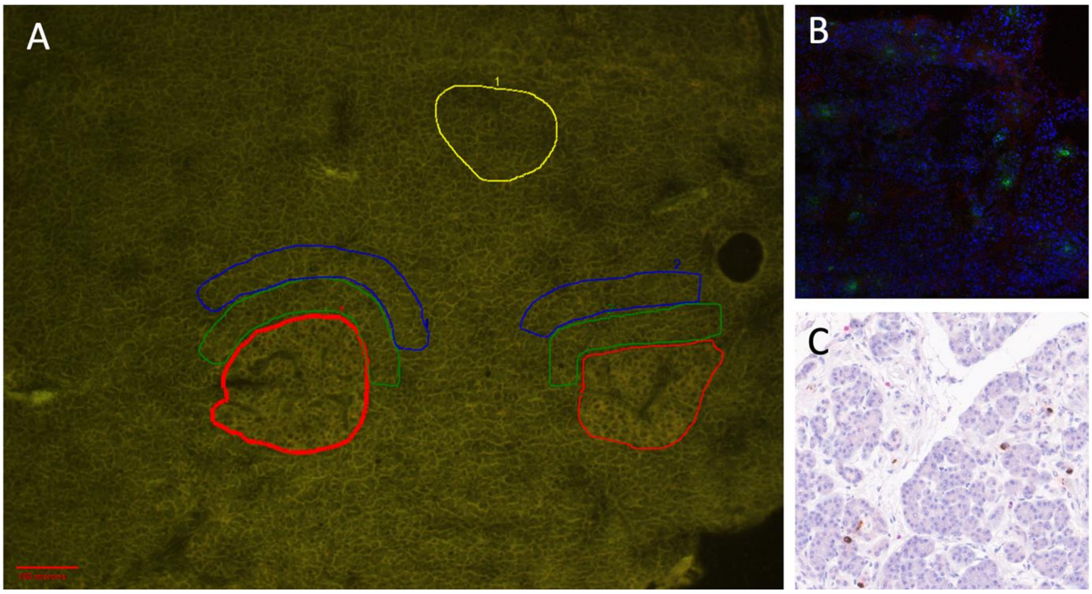

Figure S2. Auto-fluorescence of islets and marked regions before microdissection (A). All islets were insulin-negative but scattered insulin-positive cells were found within the pancreatic tissue as detected by immunofluorescence (B) and by immunohistochemistry (C). Immunohistochemistry sections were stained for insulin (guinea pig polyclonal anti-insulin, dilution 1:200, Agilent, cat: A0564) and counterstained with heamatoxylin using a standard protocol.

Red region: islets, green region: Exo1 (0-50 µm from islets), blue region: Exo2 (50-100 µm from islets) and yellow region: Exo3 (a minimum of 100 µm from islets).
